# Supplementary material for: Gluconeogenic compensation renders glycogen phosphorylase non-essential as an insecticidal target in Plutella xylostella
Source: eLife. 2026 Oct 13;15:RP111144. doi: 10.7554/eLife.111144 (PMC13661043; doi:10.7554/eLife.111144)

Figure 2–source data 2. Source data for SDS-PAGE and Western blot to Figure 2 panel A.

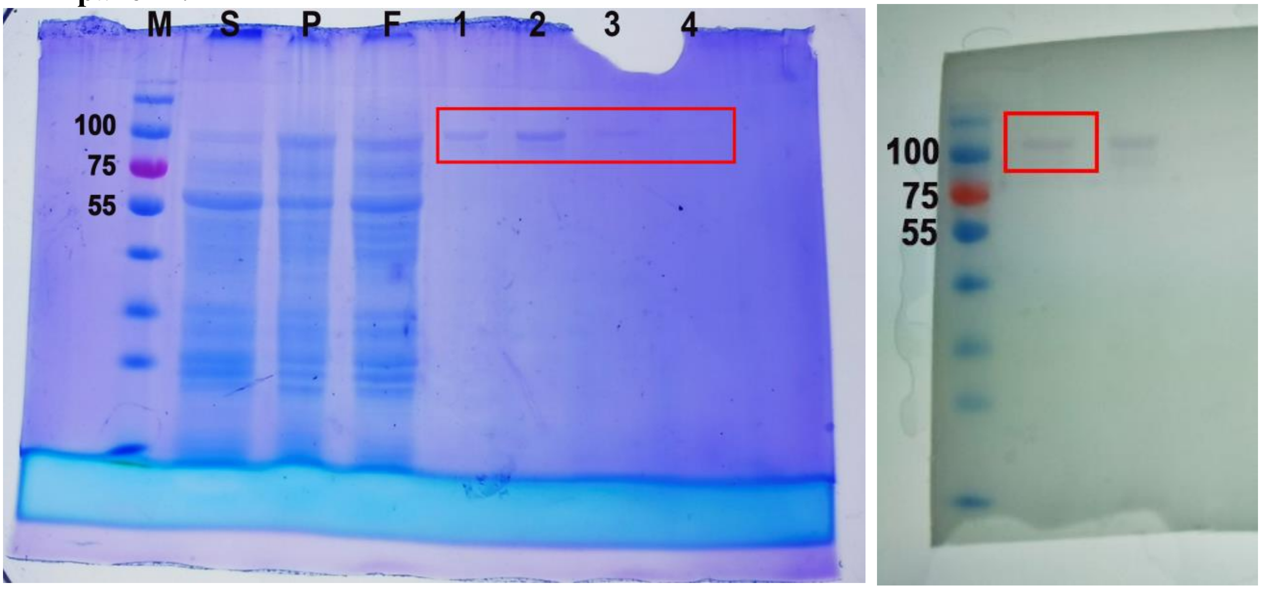

Supplement: Supplementary file 5 [file elife-111144-fig2-data2.zip › eLife-VOR-RA-2026-111144R1-Figure 2–source data 2.pdf]
