# Supplementary material for: Gluconeogenic compensation renders glycogen phosphorylase non-essential as an insecticidal target in Plutella xylostella
Source: eLife. 2026 Oct 13;15:RP111144. doi: 10.7554/eLife.111144 (PMC13661043; doi:10.7554/eLife.111144)
Supplement: Peer review history

# Editor report: eLife Assessment

**DOI:** 10.7554/eLife.111144.4.sa0

**Authors:** Hugo J Bellen

**Affiliations:** Baylor College of Medicine United States

**Keywords:** Convincing, Valuable

This study provides **valuable** evidence that glycogen phosphorylase is unlikely to represent an effective insecticidal target in Plutella xylostella and that diflubenzuron does not directly inhibit this enzyme. The combination of biochemical, molecular and physiological approaches provides **convincing** data to support these conclusions, although the proposed metabolic compensation mechanism is supported primarily by indirect evidence and would benefit from direct demonstration.

# Referee report: Reviewer #1 (Public review)

**DOI:** 10.7554/eLife.111144.4.sa1

[Editors' note: this version has been assessed by the Reviewing Editor without further input from the original reviewers.]

Summary:

In this study, the authors investigate whether glycogen phosphorylase represents a molecular target of benzoylphenylurea insecticides and evaluate the physiological consequences of suppressing glycogen phosphorylase activity in the diamondback moth Plutella xylostella. The authors combine recombinant protein biochemistry, enzyme inhibition assays, RNA interference, structural modelling, metabolite profiling, gene expression analyses, and physiological measurements to determine whether diflubenzuron directly inhibits glycogen phosphorylase and whether suppression of this enzyme is sufficient to impair insect development. Based on these experiments, the authors conclude that diflubenzuron does not directly inhibit glycogen phosphorylase and that insects tolerate substantial suppression of this enzyme through compensatory metabolic responses.

Strengths:

This study addresses an important question in insect toxicology by systematically evaluating glycogen phosphorylase as a potential insecticidal target. The authors combine complementary biochemical, molecular, physiological, and structural approaches, including recombinant enzyme characterization, inhibitor assays, RNA interference, metabolite profiling, structural modelling, and measurements of fitness-related traits. This integrative approach provides a comprehensive evaluation of the biological consequences of glycogen phosphorylase suppression. In particular, the biochemical evidence that diflubenzuron does not inhibit glycogen phosphorylase, together with the observation that strong suppression of glycogen phosphorylase produces only transient physiological effects without measurable impacts on development or reproduction, provides strong support for the conclusion that glycogen phosphorylase is unlikely to represent an effective standalone insecticidal target.

Weaknesses:

The main limitation concerns the proposed mechanism underlying metabolic compensation. The observed increases in gluconeogenic gene expression, changes in metabolite abundance, and reductions in total protein are consistent with activation of compensatory metabolism, but are insufficient to directly demonstrate increased gluconeogenic flux or establish that amino acid-derived carbon is incorporated into newly synthesized glucose. Similarly, although the analyses of glycogen-associated enzymes strengthen the discussion of alternative metabolic pathways, changes in gene expression alone do not demonstrate that these pathways contribute to glycogen utilization in vivo.

Some mechanistic interpretations therefore extend beyond the data presented. For example, decreases in total protein are interpreted as evidence of protein catabolism fuelling gluconeogenesis, yet they do not directly demonstrate amino acid mobilization or incorporation into glucose. Likewise, increased expression of gluconeogenic genes is interpreted as evidence of increased pathway activity, although transcriptional changes do not necessarily reflect metabolic flux. Finally, the absence of major developmental defects following glycogen phosphorylase suppression is attributed primarily to metabolic compensation, but an alternative explanation is not fully considered. Such explanation could be that glycogen phosphorylase is not rate-limiting for glucose homeostasis under the nutrient-rich experimental conditions, where dietary carbohydrates are continuously available. Consequently, the proposed compensatory mechanism remains plausible and well supported by indirect evidence, but several aspects would benefit from more cautious interpretation.

Overall, the authors successfully achieve their primary objective of evaluating glycogen phosphorylase as a candidate insecticidal target. The study provides useful biochemical and physiological evidence that this enzyme is unlikely to represent an effective target for insecticide development in P. xylostella, while highlighting the importance of metabolic plasticity when assessing metabolic targets. The experimental approaches and datasets presented here should be valuable to researchers studying insect metabolism, insecticide mode of action, and target validation, although the precise mechanisms underlying the proposed metabolic compensation remain an important subject for future investigation.

# Author comment: Author response

**DOI:** 10.7554/eLife.111144.4.sa2

**Authors:** Yifei Zhou, Yanqi Kang, Yan Liu, Ruichi Li, Dongliang Wang, Chong Yi, Yifan Li, Yalin Zhang, Zhen Tian, Jiyuan Liu

**Affiliations:** Northwest A&F University Yangling China; Northwest A&F University Yangling China; Northwest A&F University Yangling China; Northwest A&F University Yangling China; Northwest A&F University Yangling China; Northwest A&F University Yangling China; Northwest A&F University Yangling China; Northwest A&F University Yangling China; Northwest A&F University Yangling China; Northwest A&F University Yangling China

The following is the authors’ response to the previous reviews.

**Public Reviews:**

**Reviewer #1 (Public review):**

Strengths:

This study addresses an important question in insect toxicology by systematically evaluating glycogen phosphorylase as a potential insecticidal target. The authors combine complementary biochemical, molecular, physiological, and structural approaches, including recombinant enzyme characterization, inhibitor assays, RNA interference, metabolite profiling, structural modelling, and measurements of fitness-related traits. This integrative approach provides a comprehensive evaluation of the biological consequences of glycogen phosphorylase suppression. In particular, the biochemical evidence that diflubenzuron does not inhibit glycogen phosphorylase, together with the observation that strong suppression of glycogen phosphorylase produces only transient physiological effects without measurable impacts on development or reproduction, provides strong support for the conclusion that glycogen phosphorylase is unlikely to represent an effective standalone insecticidal target.

We thank the reviewer for the positive assessment of our study and for recognizing the value of our integrative approach, including recombinant enzyme characterization, RNAi, metabolite profiling, structural modelling, and fitness measurements. We are also grateful for the acknowledgement that our biochemical evidence and phenotypic observations provide strong support for the conclusion that GP is unlikely to be an effective standalone insecticidal target.

Weaknesses:

(1) The proposed metabolic compensation mechanism is supported by indirect evidence.

We agree with the reviewer that our data demonstrate correlation with, rather than direct proof of, increased gluconeogenic flux. We have revised the manuscript throughout to moderate our interpretations and to clearly frame the compensation model as a plausible interpretation supported by multiple lines of indirect evidence, rather than an established mechanism. Specific revisions are detailed below.

Specific revisions:

(1) Abstract (Lines 35–39):

“We provide evidence that insects compensate through a multi-layered metabolic response: upregulation of gluconeogenic enzymes (PEPCK, G-6-Pase), selective upregulation of glycogen branching enzyme (GBE) but not α-amylase, and changes in protein content suggestive of catabolic substrate mobilization”

(2) Abstract (Lines 41–42):

“Indicating that the metabolic compensation response is ultimately effective in sustaining development”

(3) Abstract (Lines 42–44):

“These findings suggest that GP is functionally non-essential under these conditions, likely through a combination of gluconeogenic compensation and the availability of alternative carbon sources”

(4) Introduction (Lines 88–90):

“Our findings reveal that PxGP is functionally non-essential for larval development under the tested conditions, reflecting a previously uncharacterized gluconeogenic compensation mechanism”

(5) Results – Gene expression (Line 265):

“Gene expression analysis is consistent with gluconeogenic activation”

(6) Results – Protein content (Line 278):

“Changes in protein content suggestive of catabolic substrate mobilization”

(7) Results – Protein decline (Line 283):

“This protein decline is consistent with substrate mobilization”

(8) Results – GBE expression (Line 389):

“*PxGP* knockdown selectively upregulates glycogen branching enzyme expression”

(9) Results – GBE differential response (Lines 400–403):

“This differential expression pattern—selective *GBE* upregulation with unchanged *α-amylase* expression—indicates that the compensatory response to GP suppression involves targeted remodeling of glycogen structure rather than a generalized upregulation of all glycogen-degrading enzymes.”

(10) Results – Fitness assessment (Lines 411–412):

“To determine whether the metabolic changes observed following *PxGP* knockdown are associated with measurable physiological consequences”

(11) Results – Fitness interpretation (Lines 426–430):

“GP suppression likely triggers protein catabolism to supply amino acids for gluconeogenesis, contributing to transient weight loss. In the presence of continuous dietary carbohydrate supply, the compensatory response appears sufficiently effective to restore metabolic homeostasis before developmentally critical transitions”

(12) Discussion – Glycogen accumulation paradox (Line 520-521):

“a paradox that could be explained by the activation of GP-independent glycogen catabolism via alternative enzymes like...”

(13) Results – Trehalose and G6P (Lines 299–303, 315–317):

“Trehalose levels remained stable at 48-72 h but increased substantially by 96 h (7.44-fold elevation, *P* < 0.05) (Figure 9F). This increase coincided with the significant upregulation of gluconeogenic genes (e.g., *PEPCK* and *G-6-Pase*), consistent with the idea that gluconeogenesis contributes to trehalose synthesis and ensures adequate carbohydrate reserves for the upcoming pupation”

And: “Together, the coordinated behavior of G6P and trehalose is consistent with gluconeogenesis-derived glucose being converted into storage and transport carbohydrates”

(14) Results – Integrated interpretation (Lines 330–332):

“Collectively, these metabolite and gene expression data reveal a biphasic metabolic adaptation that provides a coherent explanation for why substantial GP suppression causes no mortality or developmental defects”

(15) Discussion – Definitive proof (Lines 527–531):

“At 96 h, trehalose and G6P levels in ds*GP*-treated larvae were maintained at markedly higher levels than in ds*GFP* controls, which had declined by this time point, coinciding with 3–4‑fold upregulation of *PEPCK* and *G‑6‑Pase*. These changes strongly support, albeit indirectly, de novo glucose synthesis as the primary rescue mechanism.”

(16) Discussion – Protein catabolism confirmation (Lines 505):

“provides independent biochemical support for protein catabolism”

(2) Some mechanistic interpretations extend beyond the data presented.

We accept this criticism and have systematically revised the manuscript to distinguish more carefully between observed transcriptional/metabolic changes and functional interpretations. We now consistently frame these as correlative evidence consistent with—but not proving—the proposed mechanisms.

Specific revisions:

The revisions listed under Weakness 1 above—particularly those modifying language around protein decline (Lines 278, 283), gene expression (Lines 265, 389), and trehalose/G6P changes (Lines 299–303, 314–317)—also directly address this weakness by removing the implication that transcriptional or protein-level changes constitute functional proof of pathway activation. Additionally, we have made the following revisions:

(17) Additional discussion of GBE/α-amylase limitation (Line 405-408)

We have added explicit acknowledgement that transcriptional changes alone do not demonstrate functional pathway activation:

“However, as these observations are limited to the transcript level, further enzymatic activity assays or glycogen structure analyses would be required to determine whether this transcriptional change translates into functional alterations in glycogen mobilization.”

(18) Fitness section – clarification (Lines 426–430):

We have revised the interpretation of transient larval weight loss as described above, introducing “likely” and clarifying that the data are consistent with the model rather than conclusively demonstrating it.

(3) An alternative explanation for the limited phenotype is not fully considered.

We thank the reviewer for this important point. We agree that the continuous availability of dietary carbohydrates under our experimental conditions represents a plausible alternative explanation for the lack of phenotype. We have added explicit discussion of this alternative interpretation in the Abstract, Introduction, and Discussion sections.

Specific revisions:

(19) Abstract (Lines 42–44):

As shown above, we have revised the abstract to include: “These findings suggest that GP is functionally non-essential under these conditions, likely through a combination of gluconeogenic compensation and the availability of alternative carbon sources.”

(20) Introduction (Lines 88–90):

As shown above, we have revised the introduction to include: “under the tested conditions” and to acknowledge that the observed phenotype reflects a combination of factors.

(21) Discussion – Biphasic response (Lines 531–539):

We have revised the discussion of the biphasic response to moderate the causal language and acknowledge alternative interpretations:

“This biphasic response—initial metabolic stress followed by delayed (48-72 h) and robust compensation—creates a temporal buffer by 96 h and is consistent with the absence of overt phenotypes despite substantial GP suppression. This temporal coordination raises the possibility of a developmentally programmed response, potentially involving hormonal regulation, that anticipates energy demands at critical transitions. Critically, this pattern suggests a potential vulnerability window (~72 h) during which combined inhibition of glycogenolysis and gluconeogenesis might overcome compensation, although this remains speculative and would require experimental validation.”

(22) Discussion – Alternative explanation paragraph (Lines 540–547):

We have added a following paragraph explicitly addressing the alternative explanation:

“We also acknowledge that the absence of a severe phenotype may reflect an additional, non‑mutually exclusive explanation: under our experimental conditions, with continuous dietary carbohydrate availability, GP activity may not be rate‑limiting for maintaining glucose homeostasis. The observed transient larval weight loss could thus reflect both active metabolic compensation and the inherently low demand on GP for glucose supply in a feeding larva. Distinguishing between active compensation and the non‑limiting nature of GP will require future studies under nutrient‑restricted conditions or during fasting intervals, where GP's role is likely to become more critical.”

Additional revisions to moderate language throughout the manuscript

Beyond the specific revisions addressing each weakness, we have also made the following broader language modifications to ensure consistent and cautious interpretation throughout:

(23) Changes from “ablation” to “suppression” (Lines 180, 195–197, 357):

“Ablation” to “suppression” throughout.

(24) Conclusions – Fundamental principle (Lines 619):

“our findings uncover a fundamental principle of...”

(25) Conclusions – Strategic path (Lines 621–623):

“This metabolic plasticity renders GP non-viable as a standalone insecticidal target but illuminates a potential strategic path forward”

(26) Discussion – Trehalase inhibitors (Lines 556–558):

“For example, trehalase inhibitors such as Validamycin A are potent insecticides [44, 45], suggesting that trehalase inhibition may not be subject to the same degree of metabolic compensation”

(27) Discussion – Metabolic pincer (Line 561):

“a 'metabolic pincer' attack that might overcome adaptive compensation”

(28) MM/GBSA and structural predictions (Lines 27–32, 450, 455–457):

Abstract (Lines 27–32)

“Molecular docking and MM/GBSA analysis predict that this selectivity reflects differential side-chain engagement: GPI is predicted to occupy the allosteric site at the dimer interface via contacts with seven residues spanning both subunits (ΔG = −34.63 kcal/mol), whereas DFB's difluorobenzoyl moiety is predicted to remain solvent-exposed without productive protein contacts (ΔG = −29.29 kcal/mol).”

Line 450

“MM/GBSA analysis indicated”

Lines 455–457

“These structural predictions are consistent with established structure–activity relationships of acyl urea compounds [15], in which target selectivity is governed by the side-chain substitution pattern rather than by the shared acyl urea core”

(29) Scope of conclusion (Lines 471–472):

“This provides the first direct biochemical evidence excluding GP as a candidate molecular target for diflubenzuron”

**Recommendations for the authors:**

**Reviewer #1 (Recommendations for the authors):**

Major recommendations

(1) Moderate the interpretation of the proposed compensatory mechanism throughout the manuscript.

We fully agree with the reviewer's assessment. Throughout the manuscript, we have systematically moderated the language used to describe the compensatory mechanism, ensuring that our interpretations are framed as plausible inferences supported by multiple lines of indirect evidence rather than as established conclusions. The specific revisions are detailed in items (1)–(7), items (10)–(16) of our response to Weakness 1 above, and items (24)–(25) of our response to Weakness 3 above.

Additional language modifications addressing the reviewer's concern about assertive terminology are detailed in our response to Minor Recommendation 1 below.

Line 495

“Our investigation shows that this tolerance reflects a robust”

Line 498

“As insects elicited a compensatory gluconeogenic pathway.”

Line 518

“Direct metabolite quantification reflected this compensation”

(2) Clarify the evidence supporting alternative glycogen degradation pathways.

We thank the reviewer for this valuable suggestion. We have revised the GBE and α-amylase sections to more clearly distinguish between transcriptional changes and functional pathway activation. The revised wording now explicitly acknowledges that our observations are limited to the transcript level and that functional confirmation would require additional experiments. The specific revisions are detailed in items (8)–(9) and item (12) of our response to Weakness 1 above. We have also added a sentence acknowledging that these observations are limited to the transcript level (item 17) of our response to Weakness 2 above.

(3) Discuss alternative explanations for the limited physiological phenotype.

We thank the reviewer for this important point. We agree that the continuous availability of dietary carbohydrates under our experimental conditions represents a plausible alternative explanation for the lack of phenotype. We have incorporated this alternative interpretation into the Abstract, Introduction, and Discussion sections, as detailed in items (3)–(4) of our response to Weakness 1 above, and items (21)–(22) of our response to Weakness 3 above.

We have also revised the subsequent conclusion (Lines 553–554) to align with this alternative interpretation, changing “GP fails as a standalone target due to compensation via gluconeogenesis” to “GP appears to fail as a standalone target, at least in part because of compensation via gluconeogenesis.” This ensures consistency with the preceding discussion of GP’s potentially non‑rate‑limiting role.

Minor recommendations

(1) Review the manuscript for statements using terms such as "demonstrates," "confirms," "proves," or "establishes."

We have systematically reviewed the entire manuscript and replaced overly assertive terms (e.g., “demonstrates,” “confirms,” “proves,” “establishes”) with more cautious language (e.g., “suggests,” “is consistent with,” “provides evidence that,” “indicates”) wherever they refer to the proposed metabolic mechanism. Specific revisions are listed under Major Recommendation 1 above. In addition:

MM/GBSA and structural predictions (Lines 27–32, 450, 455–457): Abstract (Lines 27–32): Changed “Molecular docking and MM/GBSA analysis reveal that...” to “Molecular docking and MM/GBSA analysis predict that...”

Results (Line 450): Changed “MM/GBSA analysis confirmed” to “MM/GBSA analysis indicated.”

Results (Lines 455–457): Changed “These structural data confirm that...” to “These structural predictions are consistent with...”

Conclusion scope (Lines 471–472): Changed “This provides the first direct biochemical evidence excluding GP as a candidate molecular target for BPUs” to “This provides the first direct biochemical evidence excluding GP as a candidate molecular target for diflubenzuron.”

Changes from “ablation” to “suppression” (Lines 180, 195–197, 357): Changed “ablation” to “suppression” throughout the manuscript where referring to GP knockdown.

(2) Ensure that the distinction between changes in transcript abundance, enzyme activity, and metabolic flux is maintained consistently throughout the Results and Discussion.

We have carefully reviewed the Results and Discussion sections to ensure that we consistently distinguish between transcript abundance (measured by RT-qPCR), enzyme activity (measured by activity assays), and inferred metabolic flux (not directly measured). The revisions listed under Major Recommendations 1 and 2 above directly address this point. Key changes include:

Consistently using “transcriptional upregulation” or “expression” when referring to qPCR data, rather than “activation” or “pathway activity.”

Explicitly acknowledging that transcriptional changes do not necessarily reflect metabolic flux.

Using “is consistent with” rather than “demonstrates” when linking gene expression changes to functional outcomes.

Additional clarifications and corrections to data presentation

During the preparation of the revised manuscript, we also made several corrections and clarifications to the data presentation:

RNAi knockdown efficiency (Line 364–367): We have added a clarification that the knockdown efficiency in the cohort used for enzyme activity and fitness assays (54.66% at 48 h) was lower than that achieved in the dose–response experiment (87.59% at 48 h), reflecting batch-to-batch variation between independently injected cohorts. We have also noted that the enzyme-activity data should be interpreted against the transcript reduction measured in this same cohort.

Total protein data comparability (Lines 374–380): We have added a note clarifying that the total-protein data shown in Figure 9D and Figure 10–figure supplement 2 derive from independent experimental cohorts processed at different homogenization ratios; absolute protein concentrations are therefore not directly comparable between the two panels. Both datasets nonetheless show a transient decline of approximately 30% in total protein in ds*GP*-treated larvae within the first 72 h.

Trehalose and G6P data interpretation (Lines 304–309, 314–315, 345–353): We have revised the description of trehalose and G6P levels at 96 h to clarify that the large fold-differences primarily reflect a pronounced decline in ds*GFP* control values at this time point, rather than a net increase in absolute metabolite content in ds*GP*-treated larvae. The revised wording now indicates that GP-suppressed larvae maintain their trehalose and G6P pools at a stage when control larvae are actively depleting them.

Gene expression recovery kinetics (Lines 251–252): We have corrected the description of *PxTre* and *PxHex* expression at 96 h to state that they “returned to, and modestly exceeded, control levels” rather than “returned to near baseline levels.”

Cell line description update (Lines 644–654): In response to editorial requirements, we have updated the cell line description to include supplier authentication, mycoplasma testing status, and confirmation that the cells were used experimentally within one year of purchase.

Figure corrections:

Figure 2 legend: revised to state “maximum inhibition did not exceed 55.07 ± 7.05%” to match the main text.

Figure 4 legend: corrected normalization description from “control set to 1.0 at each time point” to “calibrated to the 24 h control sample (set to 1.0).”

Figure 6 legend: corrected reference sample from “L1 set to 1.0” to “Egg set to 1.0.”

Figure 8D: corrected y-axis label from “Adult emergency (%)” to “Adult emergence (%).”

Figure 9C: corrected in-figure label from “*G-6-P*” to “*G-6-Pase*.”

Terminology correction: We have standardized the use of “similarity” (rather than “identity”) when referring to sequence comparisons (Lines 190, 1075).

PROSITE motif name correction (Lines 181–184): We have corrected “phosphatase-pyridoxal phosphate linkage site” to “phosphorylase pyridoxal-phosphate attachment site.”

Figure 11 statistical description (Lines 1138–1142): We have revised the figure legend to specify that data in (B) and (C) are mean ± SEM of three biological replicates, while data in (D–F) are shown as box plots with sample sizes indicated, and the statistical comparison is ds*GP* vs. ds*GFP* (independent samples t‑test). These revisions do not affect any results or conclusions.

Gene expression peak description (Line 301): Changed “coincided temporally with peak expression of gluconeogenic gene” to “coincided with the significant upregulation of gluconeogenic genes” to avoid implying a single defined peak.

Minor textual corrections

We have also corrected the following textual issues:

Sentence structure (Lines 433–435): We have revised the sentence structure to correct a comma splice and clarify the logical relationship. The original sentence “To provide structural insight into the observed selectivity, GPI potently inhibits PxGP (IC_50_ = 2.96 nM) while DFB does not, we performed...” has been revised to “To provide structural insight into the observed selectivity—in which GPI potently inhibits PxGP (IC_50_ = 2.96 nM) while DFB does not—we performed...”

Additional minor corrections: We have corrected a small number of typographical errors and stylistic inconsistencies throughout the manuscript.

All of these corrections are limited to data presentation, figure labeling, and textual clarity. They do not alter any experimental results, quantitative conclusions, or the overall interpretation of the study.
